# Supplementary material for: Genetically regulated eRNA expression predicts chromatin contact frequency and reveals genetic mechanisms at GWAS loci
Source: Nat Commun. 2025 Apr 3;16:3193. doi: 10.1038/s41467-025-58023-x (PMC11968980; doi:10.1038/s41467-025-58023-x)
Supplement: Supplementary file 1 — Supplementary Information [file 41467_2025_58023_MOESM1_ESM.pdf]

SUPPLEMENTARY INFORMATION

Genetically regulated eRNA expression predicts chromatin contact frequency and reveals genetic mechanisms at GWAS loci

Michael J. Betti[1], Phillip Lin[1], Melinda C. Aldrich[1], Eric R. Gamazon[1,2]

[1]Department of Medicine, Division of Genetic Medicine, Vanderbilt University Medical Center
2525 West End Avenue, Suite 700
Nashville, TN 37203, United States

[2]Clare Hall, University of Cambridge, Cambridge, England
Herschel Rd, Cambridge CB3 9AL, United Kingdom

Correspondence to: eric.gamazon@vumc.org, michael.j.betti@vanderbilt.edu

Supplementary Figures

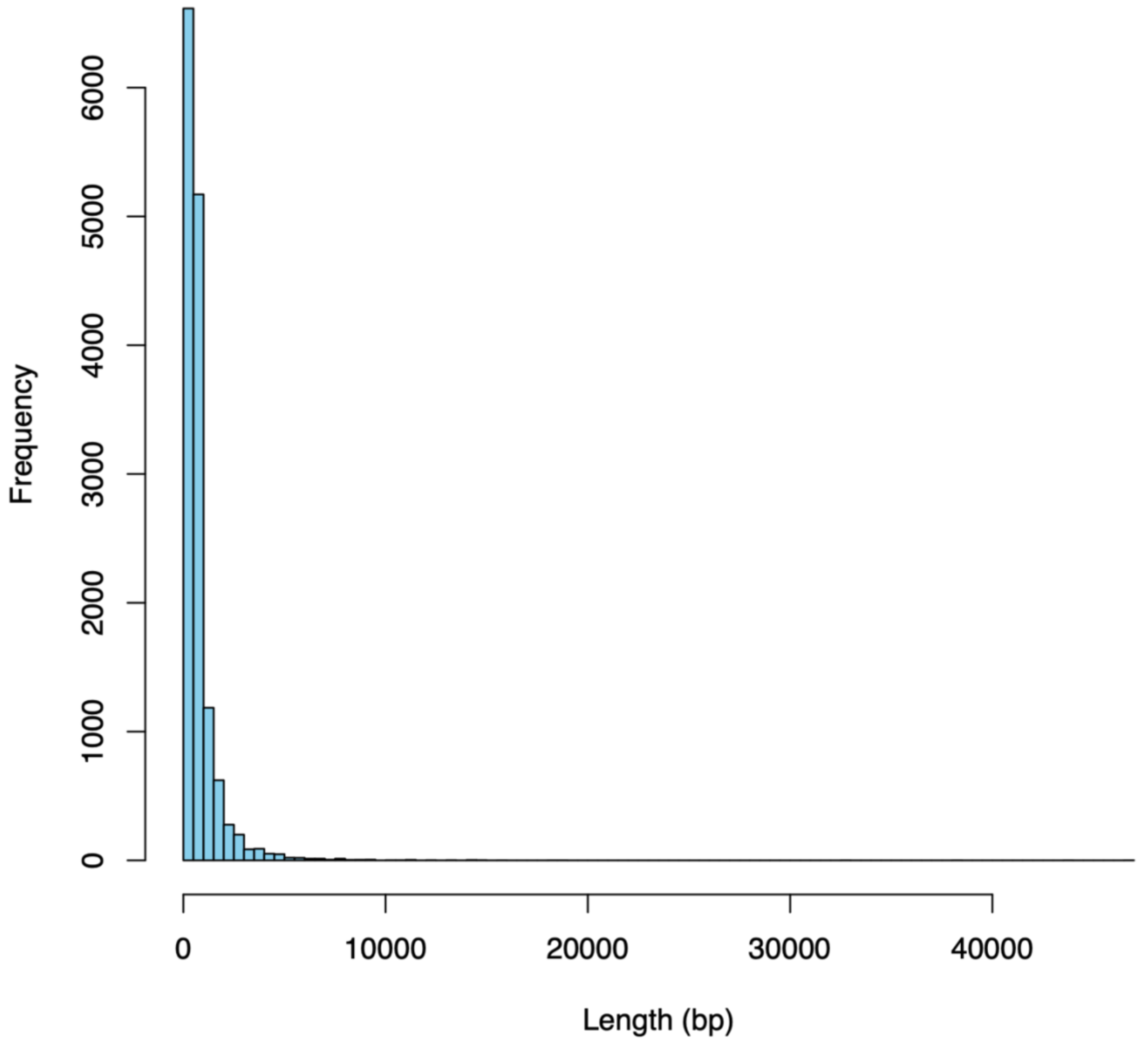

Supplementary Fig. 1. **Distribution of transcript length among the 14,471 unique eRNAs included in the GReX models.** Source data are provided as a Source Data file.

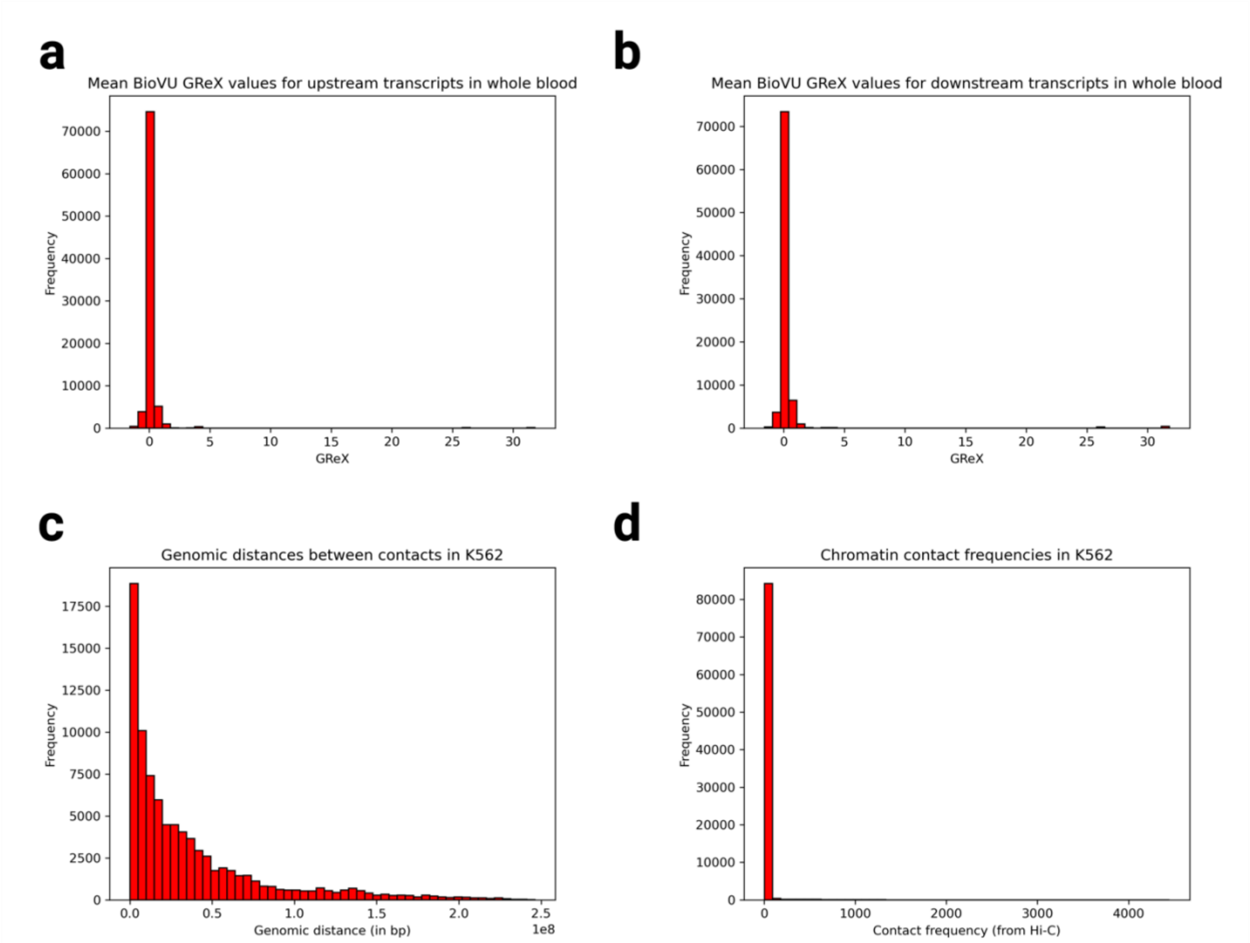

Supplementary Fig. 2. **Distributions of whole blood training and test data for the deep learning-based models of chromatin contact frequency. a** Mean genetically regulated expression (GReX) values for the upstream transcript imputed in BioVU for whole blood. **b** Mean genetically regulated expression (GReX) values for the downstream transcript imputed in BioVU for whole blood. **c** Genomic distance (in base pairs) between the transcripts in each pair. **d** Chromatin contact frequencies between whole blood transcript pairs (from Hi-C in K562). Source data are provided as a Source Data file.

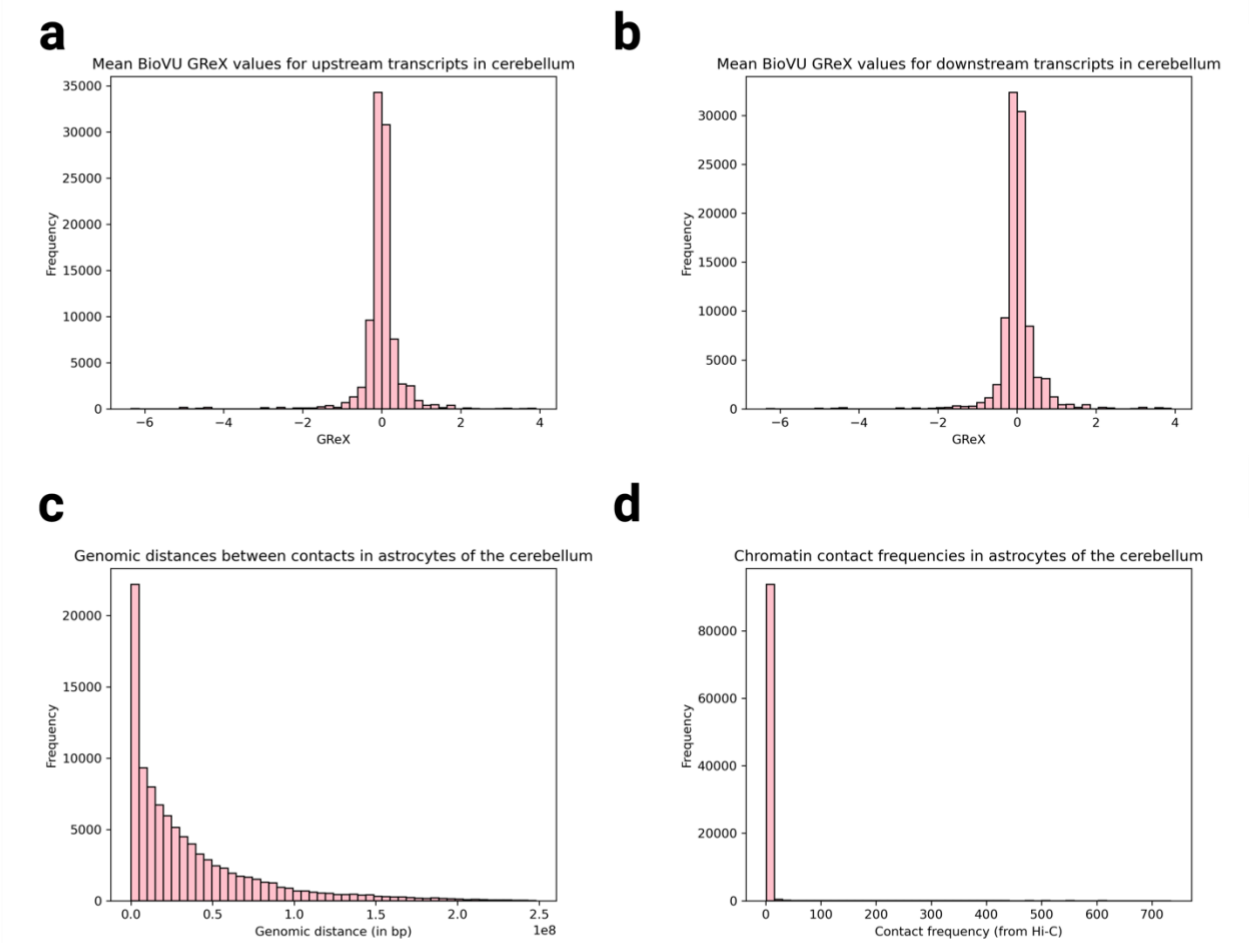

Supplementary Fig. 3. **Distributions of cerebellum test data for the deep learning-based models of chromatin contact frequency. a** Mean genetically regulated expression (GReX) values for the upstream transcript imputed in BioVU for cerebellum. **b** Mean genetically regulated expression (GReX) values for the downstream transcript imputed in BioVU for cerebellum. **c** Genomic distance (in base pairs) between the transcripts in each pair. **d** Chromatin contact frequencies between cerebellum transcript pairs (from Hi-C in astrocytes of the cerebellum). Source data are provided as a Source Data file.

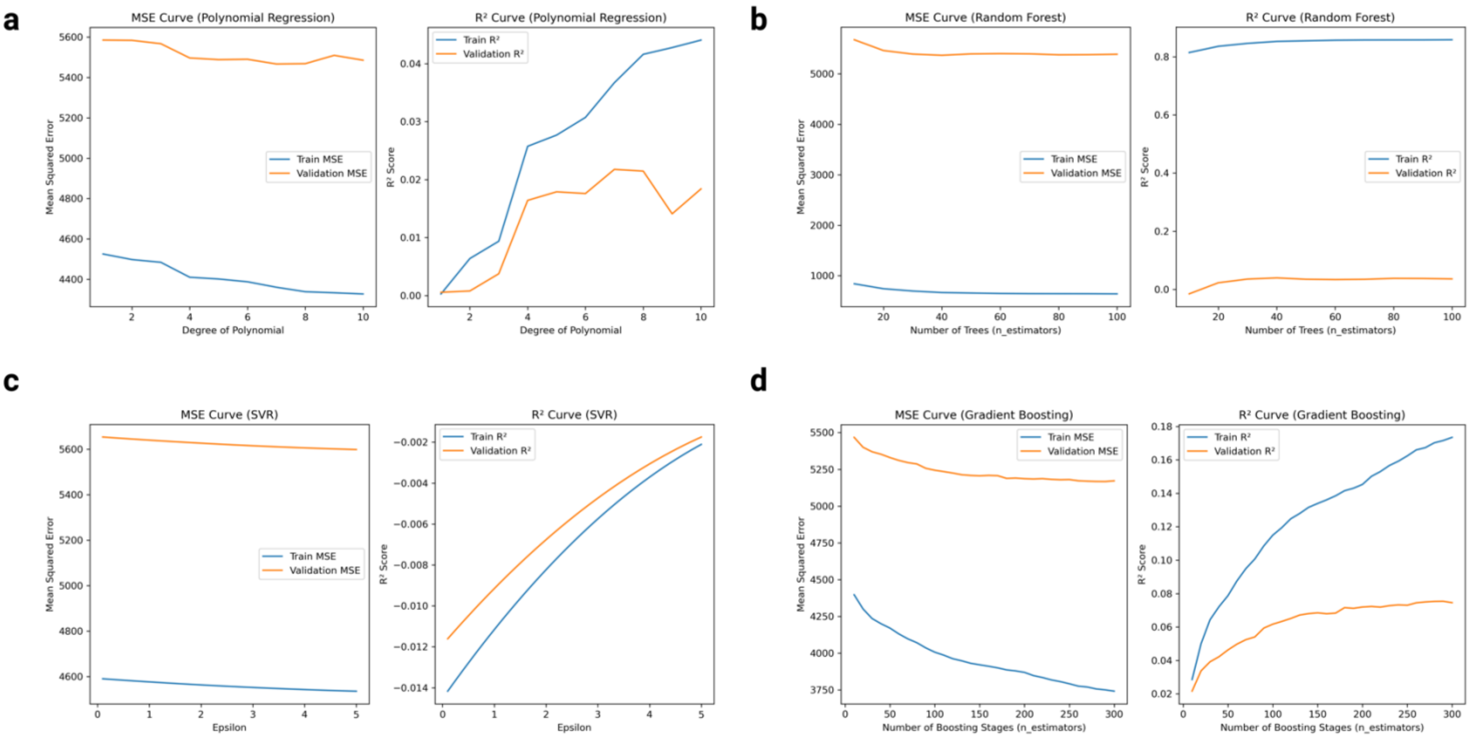

Supplementary Fig. 4. **Training curves for non-linear models in whole blood. a** We fit a series of polynomial regression models, testing a range of values for degree (1-10). The best performing model ($R^2 = 0.02$) used a degree of 7. **b** We fit a series of random forest regression models, testing different numbers of trees (10-100). The best performing model ($R^2 = 0.04$) used 40 trees. **c** We fit a series of support vector regression models, testing different values of epsilon (0.1-5.0). The best performing model ($R^2 = 0.00$) used an epsilon value of 5. **d** We fit a series of gradient boosting regression models, testing different numbers of boosting stages (10-300). The best performing model ($R^2 = 0.08$) used 290 boosting stages. Source data are provided as a Source Data file.

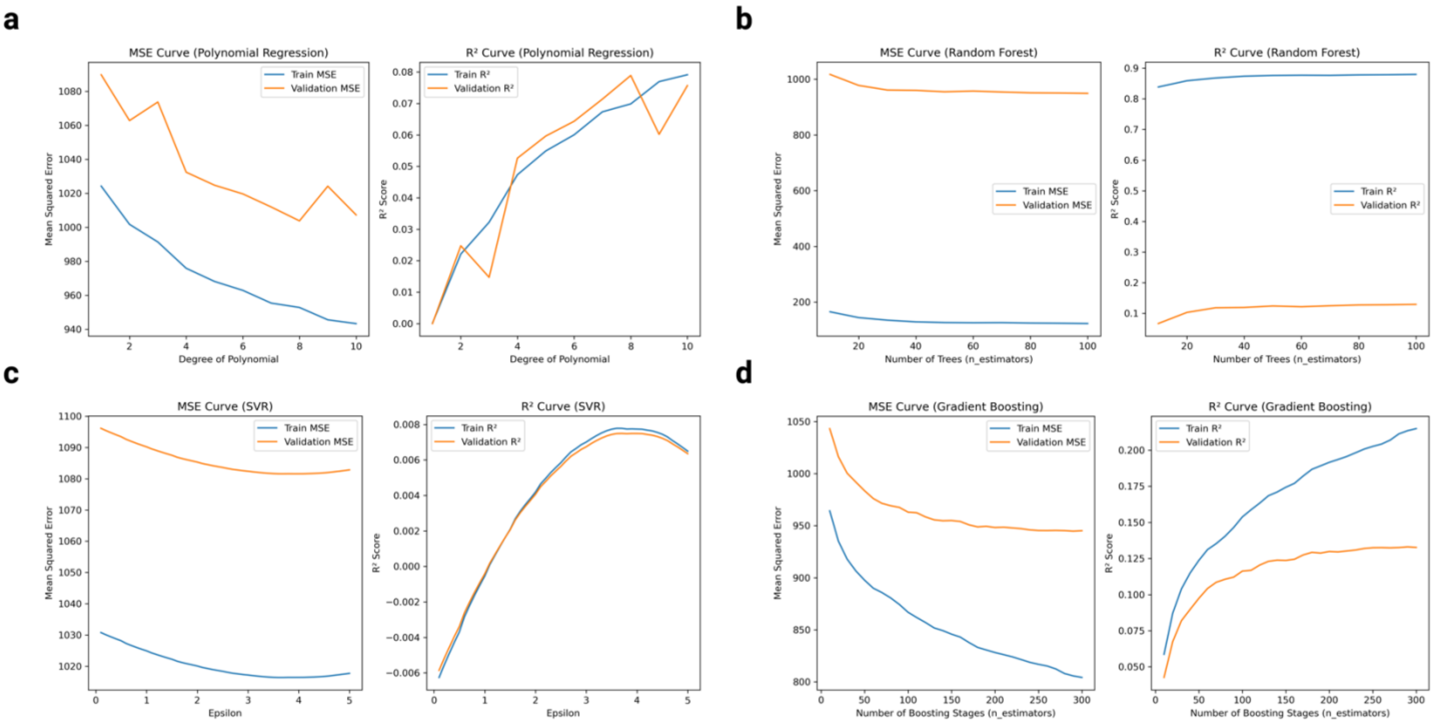

Supplementary Fig. 5. **Training curves for non-linear models in cerebellum. a** We fit a series of polynomial regression models, testing a range of values for degree (1-10). The best performing model ($R^2 = 0.08$) used a degree of 8. **b** We fit a series of random forest regression models, testing different numbers of trees (10-100). The best performing model ($R^2 = 0.13$) used 100 trees. **c** We fit a series of support vector regression models, testing different values of epsilon (0.1-5.0). The best performing model ($R^2 = 0.01$) used an epsilon value of 5. **d** We fit a series of gradient boosting regression models, testing different numbers of boosting stages (10-300). The best performing model ($R^2 = 0.13$) used 290 boosting stages. Source data are provided as a Source Data file.

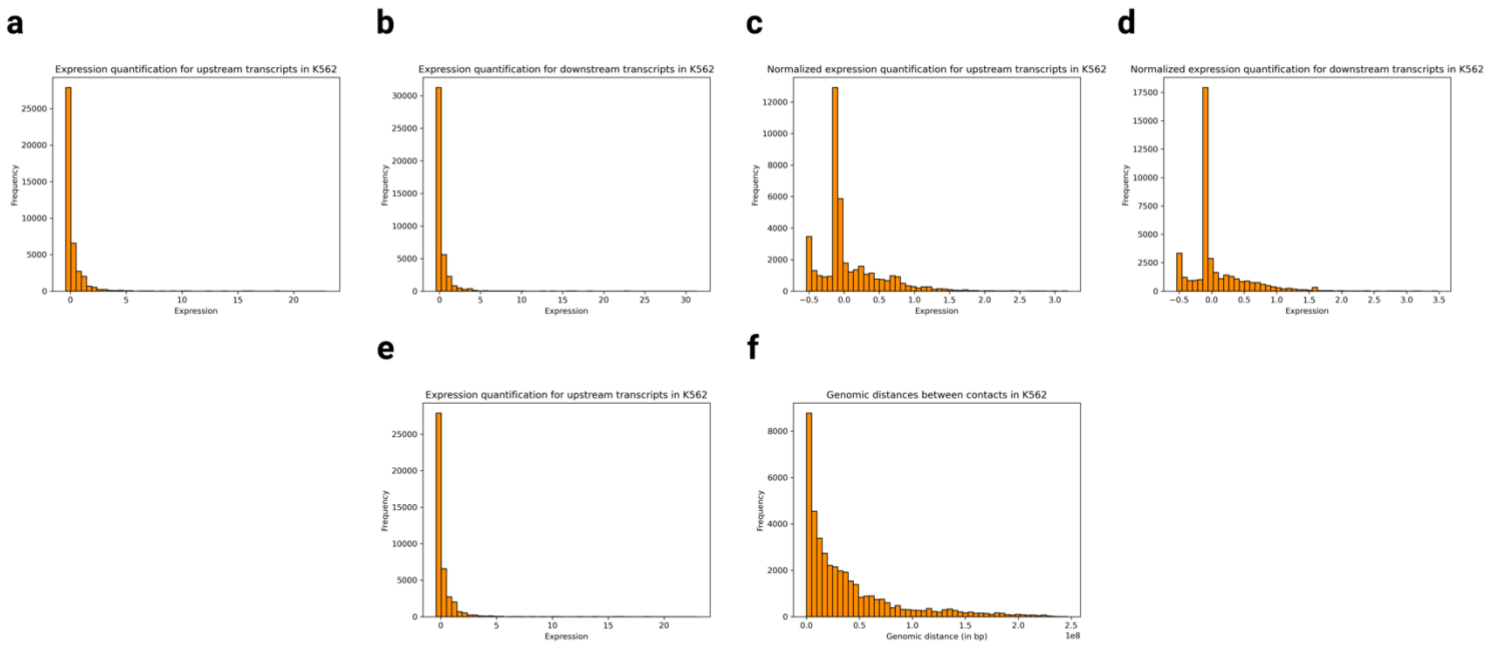

Supplementary Fig. 6. **Distributions of K562 nuclear run-on datasets for the baseline model of chromatin contact frequency. a** Expression values for the upstream transcript of each enhancer-enhancer or enhancer-gene pair prior to normalization. **b** Expression values for the downstream transcript of each enhancer-enhancer or enhancer-gene pair prior to normalization. **c** Expression values for the upstream transcript of each enhancer-enhancer or enhancer-gene pair after log1p normalization. **d** Expression values for the downstream transcript of each enhancer-enhancer or enhancer-gene pair after log1p normalization. **e** Genomic distance (in base pairs) between the transcripts in each pair. **f** Chromatin contact frequencies between cerebellum transcript pairs (from Hi-C in K562). Source data are provided as a Source Data file.

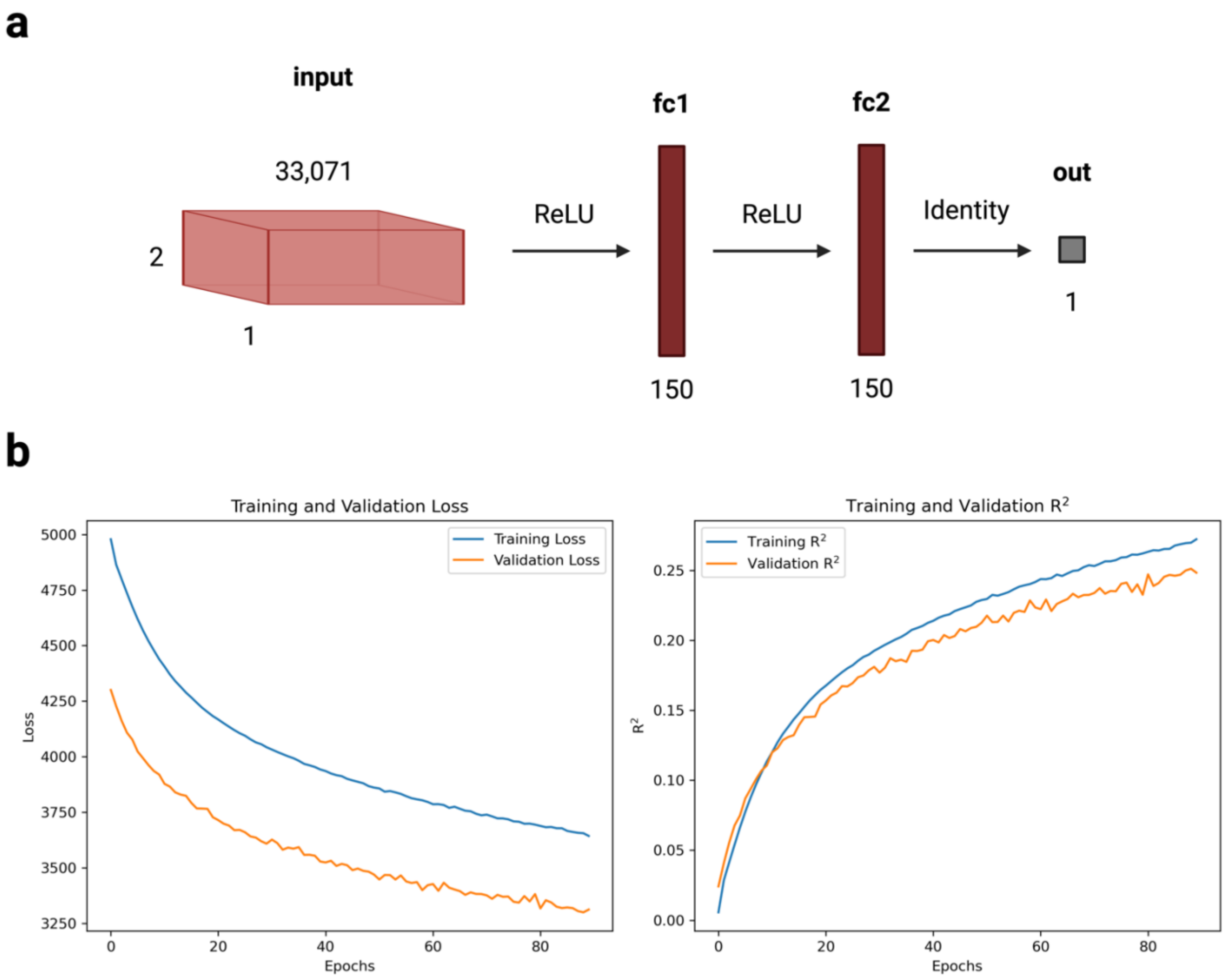

Supplementary Fig. 7. **Nuclear run-on-based baseline model. a** Grid search across 13 hyperparameters was used to achieve the optimal model architecture (see **Methods**). **b** The neural network regressor was trained for 90 epochs, achieving a mean prediction R^2 of 0.23 across the validation folds and 0.27 in the independent test set. Source data are provided as a Source Data file.

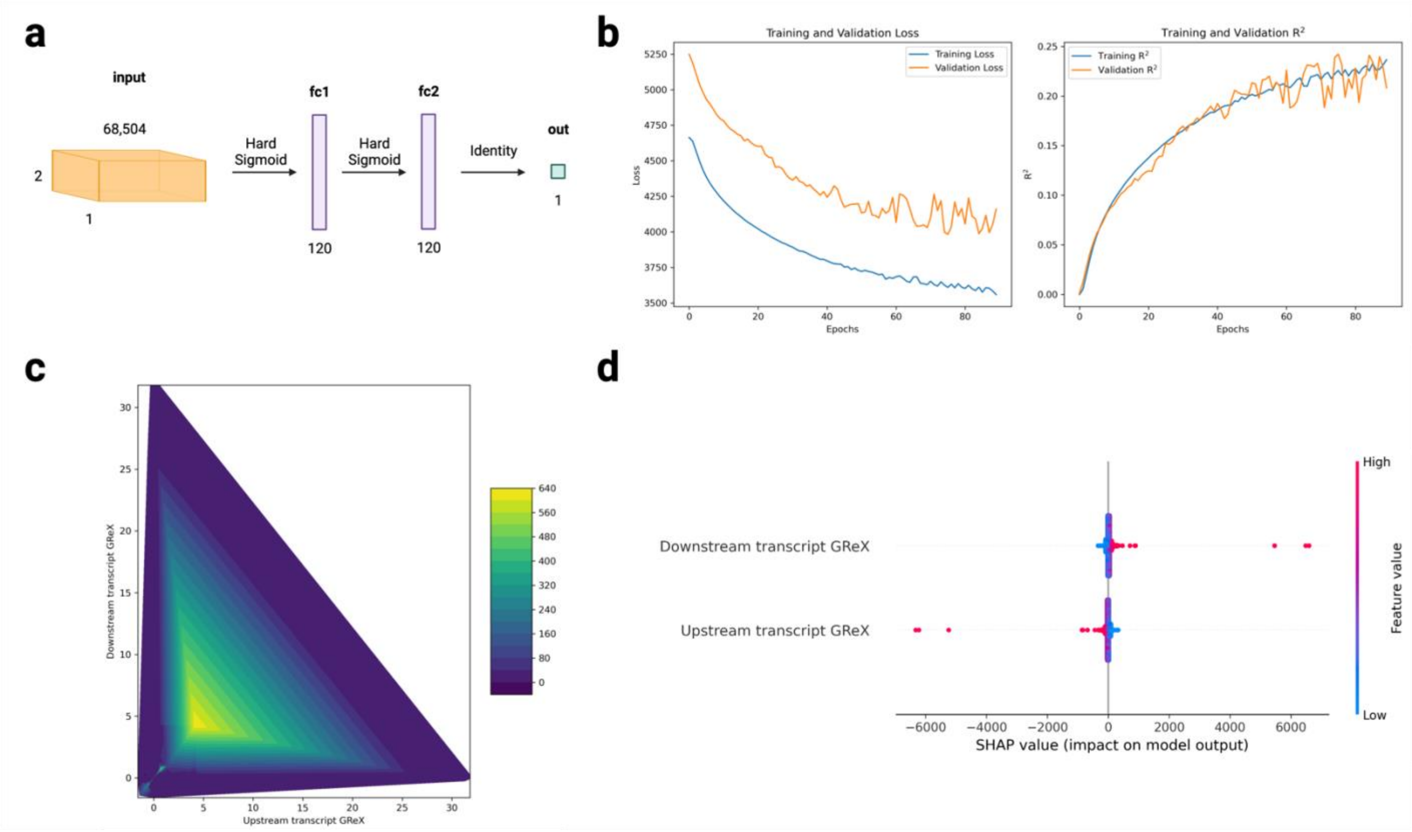

Supplementary Fig. 8. **Whole blood-based deep learning model is predictive of three-dimensional contact frequency but lacks cross-tissue portability. a** Grid search across 13 hyperparameters was used to achieve the optimal model architecture (see **Methods**). **b** The neural network regressor was trained for 90 epochs, achieving a mean prediction R^2 of 0.22 in both the validation folds and the independent test set, both of which were derived from whole blood. Within a second test tissue, cerebellum, the model achieved a prediction R^2 of 0.01. **c** Contact frequency predictions in the whole blood test set (denoted by color) relative to the input GReX of the upstream and downstream transcripts (x and y axes, respectively). **d** SHAP values computed for the transcript pairs in the test set. The relative mean contributions of the upstream and downstream transcripts were 39.33% and 60.67%, respectively. Source data are provided as a Source Data file.

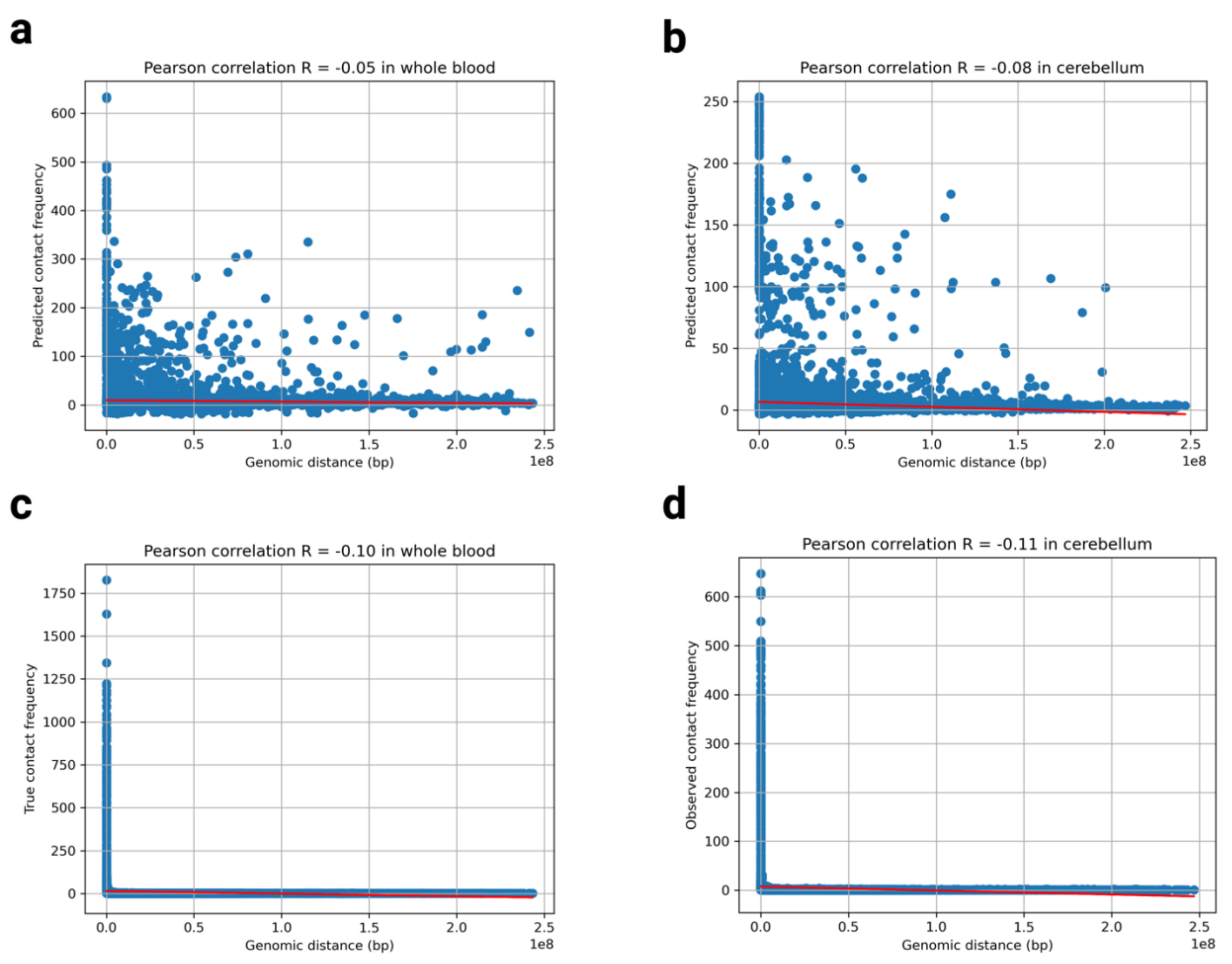

Supplementary Fig. 9. **Correlation of deep learning model predictions with linear genomic distance.** Using Pearson correlation, a weak negative correlation between genomic distance and predicted contact frequency was observed for both **a** the model trained on GReX and Hi-C data from whole blood and **b** the cerebellum-trained model. We also observed a weak negative correlation between genomic distance and observed contact frequency for both **c** the model trained on GReX and Hi-C data from whole blood and **d** the cerebellum-trained model. Source data are provided as a Source Data file.

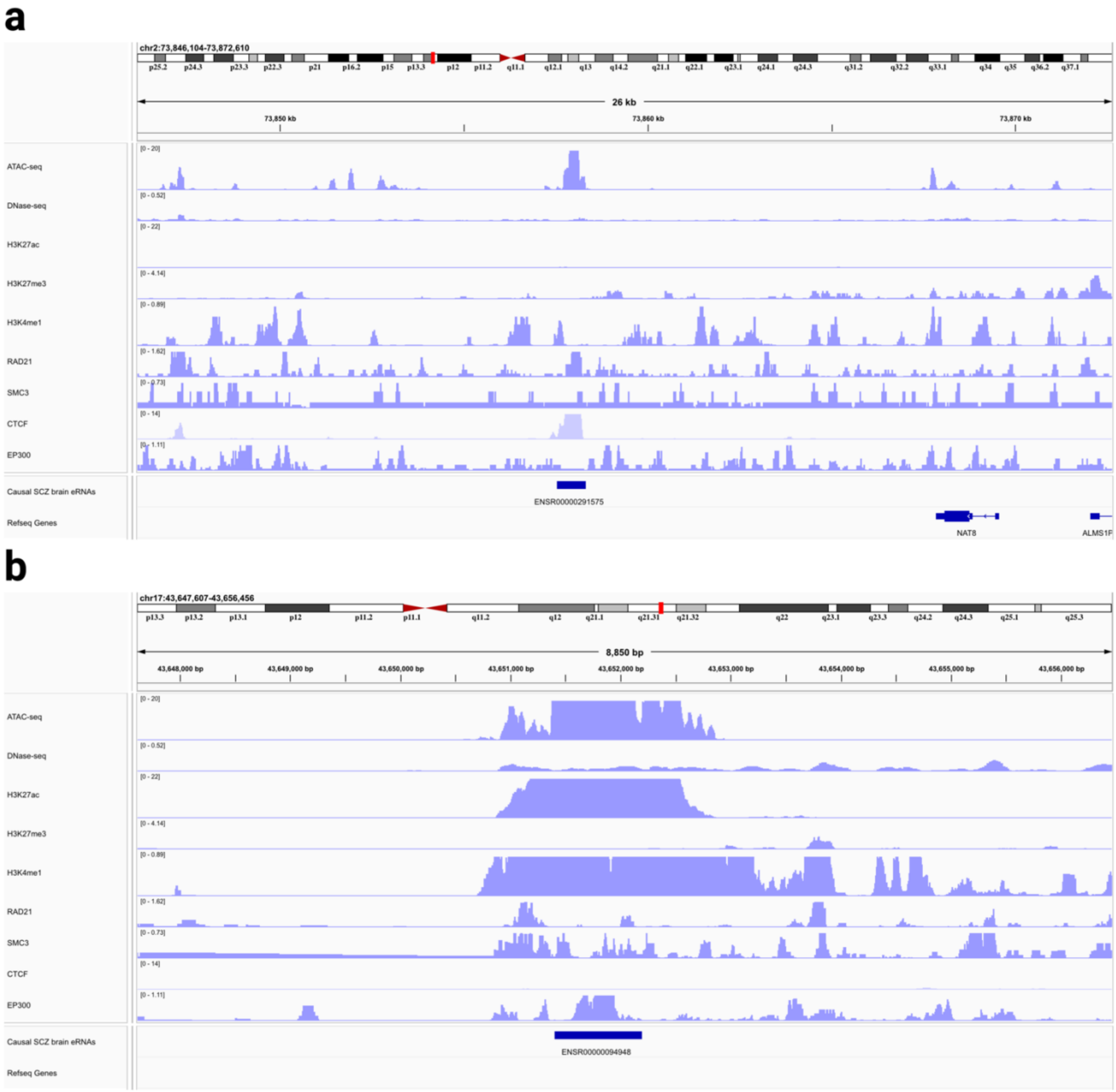

Supplementary Fig. 10. **Epigenomic patterns inform possible mechanisms by which causal eRNAs influence risk for SCZ. a** If causal eRNAs influence SCZ risk via direct mediation of enhancer-gene interactions, we would expect to observe a strong enrichment of either RAD21 and SMC3 or CTCF. However, only a small proportion of eRNAs were enriched for these features, and those that were showed no evidence of contact with a causal gene. **b** If causal eRNA expression plays a role in maintaining an open chromatin state, we should expect to observe an enrichment of ATAC-seq and DNase-seq peaks, as well as EP300 and H3K27ac, in addition to a depletion of H3K27me3. We observed enrichment for at least one of these marks in 74% of causal SCZ-associated eRNAs in the brain.

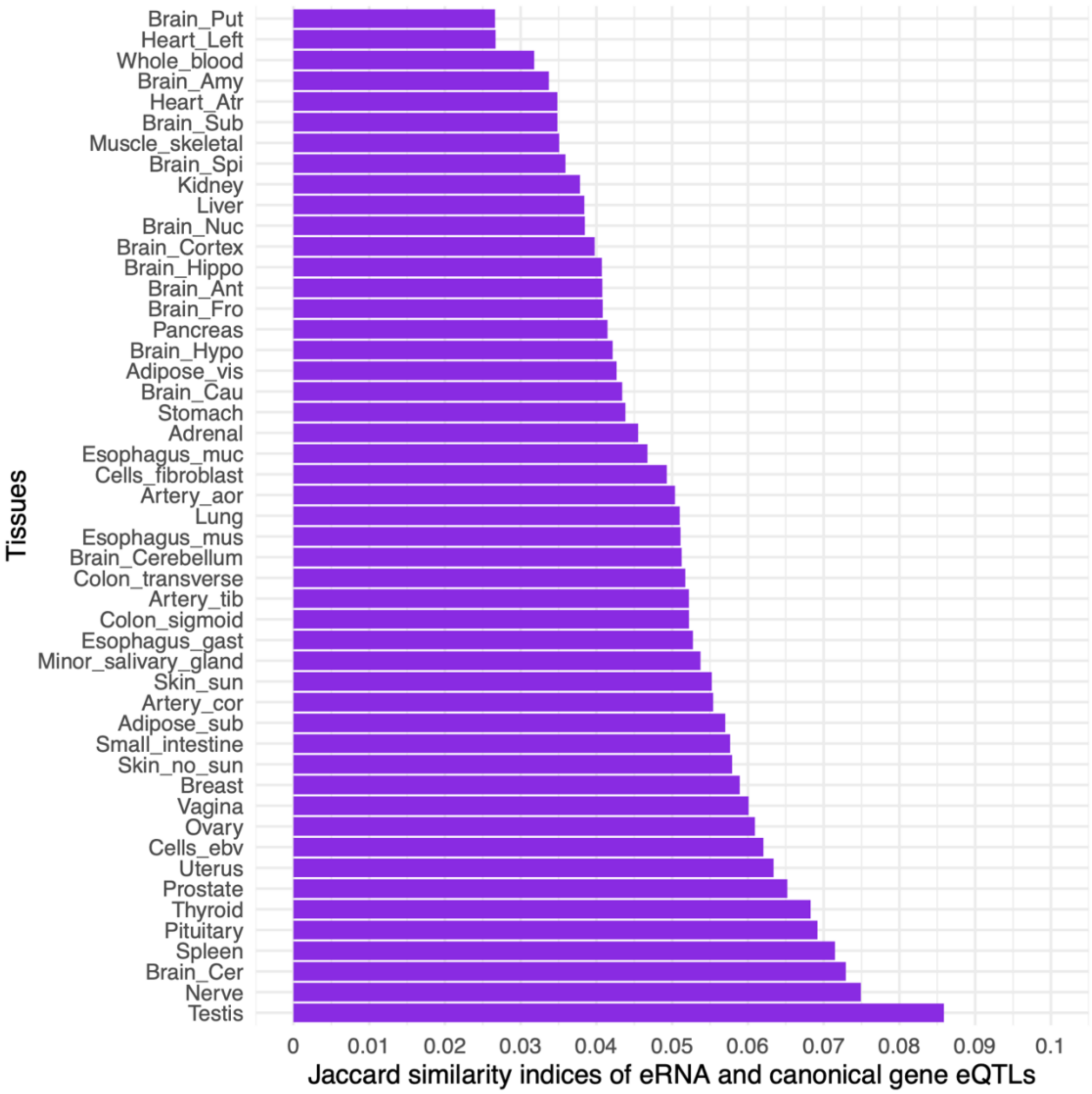

Supplementary Fig. 11. **Jaccard similarity indices assessing overlap between eRNA eQTLs and canonical gene eQTLs.** Across all tissues (n = 49), we observed a mean Jaccard similarity index of 0.05, indicating minimal overlap between eRNA and canonical gene eQTLs. The lowest Jaccard statistic was observed in brain putamen (0.03), while the highest was observed in testis (0.09). Source data are provided as a Source Data file.

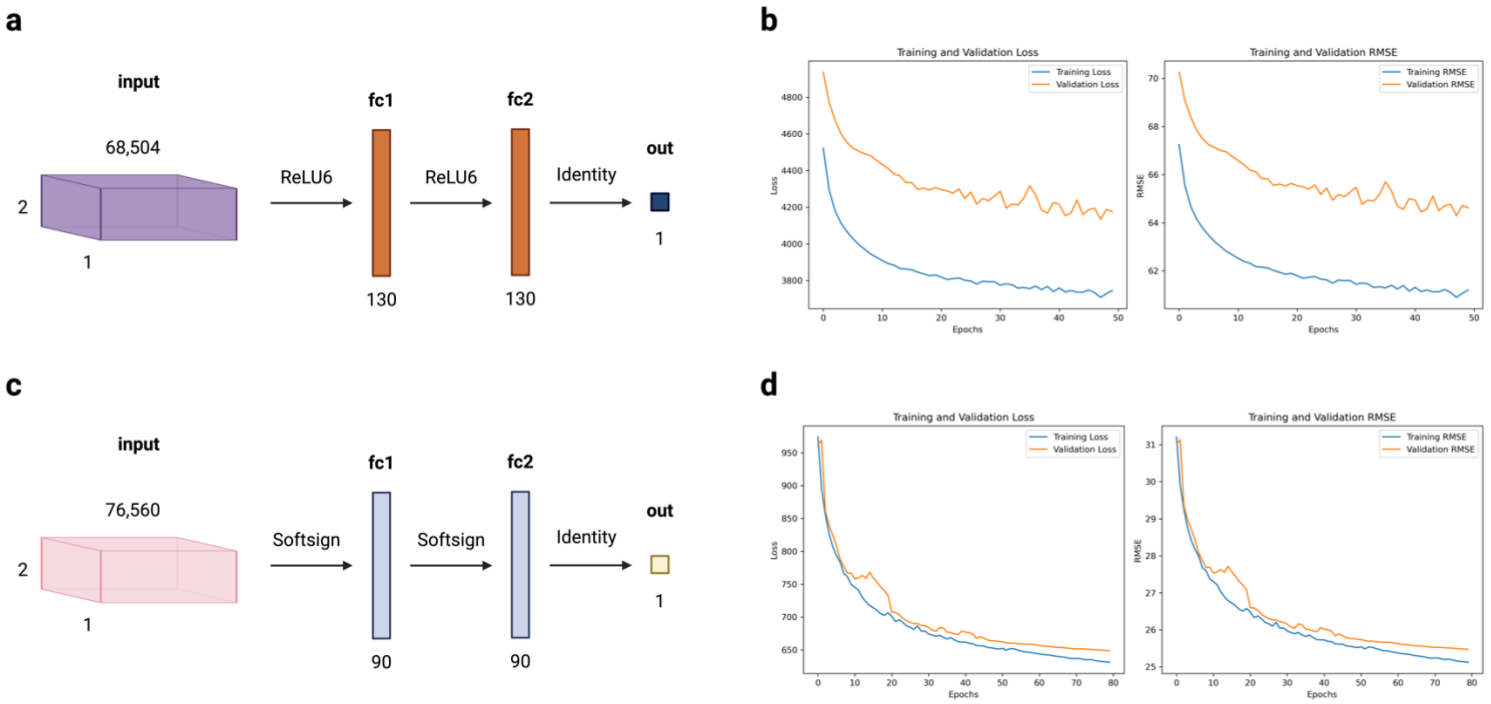

Supplementary Fig. 12. **Training neural GReX-based neural network models using root mean squared error (RMSE) as the selection criterion. a** The optimal model for whole blood consisted of two hidden layers, each with a size of 130. The ReLU6 activation function was used in the hidden layers. Weights in the hidden layers were initialized using a Kaiming uniform distribution, while those in the output layer were initialized with zeros. **b** The model was trained for 50 epochs using a batch size of 60. The NAdam optimizer was implemented with a learning rate of 0.002. The optimal model achieved a RMSE of 61.80 in the validation set, which corresponds with an R^2 of 0.20. The model that used R^2 as the selection criterion achieved a comparable validation R^2 of 0.22. **c** The optimal model for cerebellum consisted of two hidden layers, each with a size of 90. The Softsign activation function was used in the hidden layers. Weights in the hidden layers were initialized using a normal distribution, while those in the output layer were initialized with zeros. **d** The model was trained for 80 epochs using a batch size of 120. The Adagrad optimizer was implemented with a learning rate of 0.2. The optimal model achieved a RMSE of 25.47 in the validation set, which corresponds with an R^2 of 0.39. The model that used R^2 as the selection criterion achieved a comparable validation R^2 of 0.37. Source data are provided as a Source Data file.
