# Supplementary material for: Genetically regulated eRNA expression predicts chromatin contact frequency and reveals genetic mechanisms at GWAS loci
Source: Nat Commun. 2025 Apr 3;16:3193. doi: 10.1038/s41467-025-58023-x (PMC11968980; doi:10.1038/s41467-025-58023-x)
Supplement: Supplementary file 2 — Description of Additional Supplementary Files [file 41467_2025_58023_MOESM2_ESM.pdf]

DESCRIPTION OF ADDITIONAL SUPPLEMENTARY FILES

Supplementary Data 1. **Transcript lengths of the 14,471 unique enhancers included in GReX models.** Columns correspond to chromosome, start, and end coordinates of the transcript (based on hg19); transcript ID and type; and length (in bp).

Supplementary Data 2. **Whole blood test set results for the neural network-based chromatin contact frequency model.** This table contains 17,126 enhancer-enhancer and enhancer-gene contact pairs from the test set, along with their corresponding true contact frequency, predicted contact frequency, residual, and fractional error.

Supplementary Data 3. **Brain cerebellum test set results for the neural network-based chromatin contact frequency model.** This table contains 19,141 enhancer-enhancer and enhancer-gene contact pairs from the test set, along with their corresponding true contact frequency, predicted contact frequency, residual, and fractional error.

Supplementary Data 4. **Putative causal, genome-wide significant eRNA associations outside of the MHC region across all 49 cell and tissue types.** All associations were genome-wide significant in the TWAS ($p < 1.23 \times 10^{-6}$) and inferred to be causal using MR, totaling 222 eRNA-tissue pairs.

Supplementary Data 5. **Putative causal, genome-wide significant canonical gene associations outside of the MHC region across all 49 cell and tissue types.** All associations were genome-wide significant in the TWAS ($p < 1.45 \times 10^{-7}$) and inferred to be causal using MR, totaling 1,297 canonical gene-tissue pairs.

Supplementary Data 6. **Causal SCZ-associated eRNAs and canonical genes in the brain based on chromatin contacts in astrocytes of the cerebellum.** Sub-tables include pairs of causal eRNAs and causal canonical genes in 3D contact with each other (5-1), causal eRNAs not in contact with a causal gene (5-2), and causal canonical genes not in contact with a causal eRNA (5-3).

Supplementary Data 7. **TF binding motif enrichment within causal SCZ-associated eRNAs in brain.** Table includes the raw TF binding motif enrichment results using a log-likelihood ratio test implemented in FIMO[1].

Supplementary Data 8. **We identified motifs for 135 unique transcription facors that were significantly (FDR < 0.5) enriched in causal SCZ-associated eRNAs.** Table includes a list of all 135 TFs.

Supplementary Data 9. **All 88,348 genome-wide significant ($p < 2.60 \times 10^{-10}$) eRNA-tissue TWAS associations across all 4,671 UK Biobank traits**[2].

Supplementary Data 10. **Number of significant eRNA TWAS associations for each phenotype in the UK Biobank and p-values of each trait's top association.**

Supplementary Data 11. **Number of significant eRNA associations in the UK Biobank**[2] **broken down by tissue type.**

Supplementary Data 12. **Perturbed eRNAs with their corresponding TWAS associations in the UK Biobank[2].**

Supplementary Data 13. **Unique perturbed eRNAs and their corresponding linked gene.**

Supplementary Data 14. **Colocalization results for genome-wide significant ($p < 5 \times 10^{-8}$) GWAS associations in the UK Biobank and eRNA eQTLs.** Each row contains an independent, genome-wide significant phenotype-locus association from the UK Biobank. The final column ("is_colocalized") indicates whether each association has a posterior probability >= 0.7 in at least one tissue of colocalizing with an eRNA eQTL. The remaining columns contain the individual posterior probabilities of colocalization in each tissue. Colocalization was performed using the Bayesian coloc framework[3].

Supplementary Data 15. **Colocalization results for genome-wide significant ($p < 5 \times 10^{-8}$) GWAS associations in the UK Biobank and canonical gene eQTLs.** Each row contains an independent, genome-wide significant phenotype-locus association from the UK Biobank. The final column ("is_colocalized") indicates whether each association has a posterior probability >= 0.7 in at least one tissue of colocalizing with a canonical gene eQTL. The remaining columns contain the individual posterior probabilities of colocalization in each tissue. Colocalization was performed using the Bayesian coloc framework[3].

Supplementary Data 16. **QC metrics for the whole blood and cerebellum Hi-C datasets downloaded from the 4D Nucleome[4] Data Portal.**
