# Supplementary material for: Genetically regulated eRNA expression predicts chromatin contact frequency and reveals genetic mechanisms at GWAS loci
Source: Nat Commun. 2025 Apr 3;16:3193. doi: 10.1038/s41467-025-58023-x (PMC11968980; doi:10.1038/s41467-025-58023-x)
Supplement: Supplementary file 19 — Reporting Summary [file 41467_2025_58023_MOESM19_ESM.pdf]

Corresponding author(s): Michael J. Betti and Eric R. Gamazon

Last updated by author(s): Dec 24, 2024

Reporting Summary

Nature Portfolio wishes to improve the reproducibility of the work that we publish. This form provides structure for consistency and transparency in reporting. For further information on Nature Portfolio policies, see our Editorial Policies and the Editorial Policy Checklist.

Statistics

For all statistical analyses, confirm that the following items are present in the figure legend, table legend, main text, or Methods section.

n/a	Confirmed	
☐	☒	The exact sample size (*n*) for each experimental group/condition, given as a discrete number and unit of measurement
☒	☐	A statement on whether measurements were taken from distinct samples or whether the same sample was measured repeatedly
☐	☒	The statistical test(s) used AND whether they are one- or two-sided<br>*Only common tests should be described solely by name; describe more complex techniques in the Methods section.*
☐	☒	A description of all covariates tested
☐	☒	A description of any assumptions or corrections, such as tests of normality and adjustment for multiple comparisons
☐	☒	A full description of the statistical parameters including central tendency (e.g. means) or other basic estimates (e.g. regression coefficient) AND variation (e.g. standard deviation) or associated estimates of uncertainty (e.g. confidence intervals)
☐	☒	For null hypothesis testing, the test statistic (e.g. *F*, *t*, *r*) with confidence intervals, effect sizes, degrees of freedom and *P* value noted<br>*Give P values as exact values whenever suitable.*
☒	☐	For Bayesian analysis, information on the choice of priors and Markov chain Monte Carlo settings
☒	☐	For hierarchical and complex designs, identification of the appropriate level for tests and full reporting of outcomes
☐	☒	Estimates of effect sizes (e.g. Cohen's *d*, Pearson's *r*), indicating how they were calculated

Our web collection on statistics for biologists contains articles on many of the points above.

Software and code

Policy information about availability of computer code

Data collection	No software was used to collect data for this study.
Data analysis	All custom code associated with this study is publicly available on GitHub at https://github.com/mjbetti/erna-grex. This study also utilizes code from the following open source tools/repositories: bedtools (version 2.30.0), scikit-learn (version 1.2.2), PyTorch (version 1.13.1), S-PrediXcan, MR-JTI, FIMO (version 5.4.1), Matrix eQTL (version 2.3), and coloc (version 5.2.3).

For manuscripts utilizing custom algorithms or software that are central to the research but not yet described in published literature, software must be made available to editors and reviewers. We strongly encourage code deposition in a community repository (e.g. GitHub). See the Nature Portfolio guidelines for submitting code & software for further information.

Data

Policy information about availability of data

All manuscripts must include a data availability statement. This statement should provide the following information, where applicable:

- Accession codes, unique identifiers, or web links for publicly available datasets
- A description of any restrictions on data availability
- For clinical datasets or third party data, please ensure that the statement adheres to our policy

All data generated in this study (i.e. trained PrediXcan models, TWAS results, and Mendelian randomization results) are publicly available on Zenodo under the

accession code 14027849 (https://doi.org/10.5281/zenodo.14027849). The trained contact frequency models are deposited on GitHub (https://github.com/mjbetti/erna-grex) and Zenodo under accession code 14557414 (https://doi.org/10.5281/zenodo.14557414).

Research involving human participants, their data, or biological material

Policy information about studies with human participants or human data. See also policy information about sex, gender (identity/presentation), and sexual orientation and race, ethnicity and racism.

Reporting on sex and gender	The sample of BioVU individuals used in this study (n = 72,828) consisted of 31,861 males, 40,584 females, and 383 individuals with unknown sex. Gender was determined based on self-reporting and was not considered in study design, as the objective was to impute mean GReX across an entire population-level sample, irrespective of sex or gender.<br><br>TWAS in this work were performed using UK Biobank GWAS summary statistics previously generated by the Neale Lab (http://www.nealelab.is/uk-biobank/). These GWAS included both males and females across all phenotypes tested.
Reporting on race, ethnicity, or other socially relevant groupings	The sample of BioVU individuals used in this study consisted of individuals of European ancestry. The purpose of limiting to European ancestry individuals was to maintain consistency between the allele frequencies of SNPs in the trained GReX models, which were trained on the mostly European GTEx V8 dataset, and the BioVU target dataset. Genetic ancestry was inferred using principal components. Using this approach, individuals were classified based on genetic similarity to individuals from the 1000 Genomes reference populations.<br><br>The UK Biobank GWAS that were used to perform TWAS were also all based on European ancestry individuals.
Population characteristics	Among BioVU individuals, age ranged from 0-90. The median age among the cohort was 56.
Recruitment	Patients at Vanderbilt University Medical Center (VUMC) have a choice to donate any leftover samples to BioVU by signing the BioVU consent form during check-in at a VUMC outpatient clinic. Participation in BioVU is completely voluntary, and participants may opt out at any time.
Ethics oversight	The use of BioVU data has been reviewed and approved by a VUMC institutional review board (IRB 151187 and IRB 160372). Because BioVU data are de-identified, research with these samples is designated non-human subjects research.

Note that full information on the approval of the study protocol must also be provided in the manuscript.

Field-specific reporting

Please select the one below that is the best fit for your research. If you are not sure, read the appropriate sections before making your selection.

☒ Life sciences ☐ Behavioural & social sciences ☐ Ecological, evolutionary & environmental sciences

For a reference copy of the document with all sections, see nature.com/documents/nr-reporting-summary-flat.pdf

Life sciences study design

All studies must disclose on these points even when the disclosure is negative.

Sample size	The BioVU samples (n = 72,828) used in this work consisted of all individuals of European ancestry that were genotyped on the Illumina MEGA array. Because these data were used to derive mean imputed GReX values at the population level, power was not a concern.
Data exclusions	All BioVU individuals of non-European ancestries were excluded from the analyses. This was due to the GReX models being trained on the GTEx V8 dataset, which consisted of primarily European ancestry individuals. Performance of these models likely would have been reduced if the genetic ancestry of the target sample was not concordant with that of the training set.
Replication	To reproduce our experimental finding that GReX is predictive of chromatin contact frequency, we confirmed this result in two independent tissue types. We also found that an analogous model trained using nuclear run-on data showed similar performance. Finally, a model trained to predict contact frequency using GReX retained predictive performance when applied to a nuclear run-on dataset.
Randomization	This is not relevant to our study, as no group allocation was performed for this work. All TWAS that were performed utilized previously published GWAS summary statistics.
Blinding	This is not relevant to our study, as no group allocation was performed for this work.

Reporting for specific materials, systems and methods

We require information from authors about some types of materials, experimental systems and methods used in many studies. Here, indicate whether each material, system or method listed is relevant to your study. If you are not sure if a list item applies to your research, read the appropriate section before selecting a response.

Materials & experimental systems

n/a	Involved in the study
☒	☐ Antibodies
☒	☐ Eukaryotic cell lines
☒	☐ Palaeontology and archaeology
☒	☐ Animals and other organisms
☒	☐ Clinical data
☒	☐ Dual use research of concern
☒	☐ Plants

Methods

n/a	Involved in the study
☒	☐ ChIP-seq
☒	☐ Flow cytometry
☒	☐ MRI-based neuroimaging

Plants

Seed stocks	Not applicable
Novel plant genotypes	Not applicable
Authentication	Not applicable
